# Impact of STAT3 phosphorylation in glioblastoma stem cells radiosensitization and patient outcome

**DOI:** 10.18632/oncotarget.23374

**Published:** 2017-12-16

**Authors:** Konstantin Masliantsev, Baptiste Pinel, Anaïs Balbous, Pierre-Olivier Guichet, Gaëlle Tachon, Serge Milin, Julie Godet, Mathilde Duchesne, Antoine Berger, Christos Petropoulos, Michel Wager, Lucie Karayan-Tapon

**Affiliations:** ^1^ Inserm U1084, Laboratoire de Neurosciences Expérimentales et Cliniques, Poitiers F-86073, France; ^2^ Université de Poitiers, Poitiers F-86073, France; ^3^ CHU de Poitiers, Laboratoire de Cancérologie Biologique, Poitiers F-86022, France; ^4^ CHU de Poitiers, Service d’Oncologie Radiothérapique, Poitiers F-86021, France; ^5^ CHU de Poitiers, Service d’Anatomo-Cytopathologie, Poitiers F-86021, France; ^6^ CHU de Poitiers, Service de Neurochirurgie, Poitiers F-86021, France

**Keywords:** glioblastoma, Stat3, radioresistance, cancer stem cells, static

## Abstract

Glioblastoma (GBM) represents the most common and lethal primary malignant brain tumor. The standard treatment for glioblastoma patients involves surgical resection with concomitant radio and chemotherapy. Despite today’s clinical protocol, the prognosis for patients remains very poor with a median survival of 15 months. Tumor resistance and recurrence is strongly correlated with a subpopulation of highly radioresistant and invasive cells termed Glioblastoma Stem Cells (GSCs). The transcription factor STAT3 has been found to be constitutively activated in different tumors including GBM and enhanced tumor radioresistance. In this study, we assessed radiosensitization of GSC lines isolated from patients by inhibition of STAT3 activation using Stattic or WP1066. We showed that inhibitor treatment before cell irradiation decreased the surviving fraction of GSCs suggesting that STAT3 inhibition could potentiate radiation effects. Finally, we investigated STAT3 activation status on 61 GBM clinical samples and found a preferential phosphorylation of STAT3 on Serine727 (pS727). Moreover, we found that pS727 was associated with a significant lower overall patient survival and progression-free survival but not pY705. Taken together, our results suggest that pS727-STAT3 could be a potential prognostic marker and could constitute a therapeutic target to sensitize highly radioresistant GSCs.

## INTRODUCTION

Glioblastoma (GBM) is the most common and aggressive primary malignant brain tumor associated with a poor prognosis. Surgical resection followed by concomitant radiochemotherapy constitutes the gold standard treatment for glioblastoma patients [1]. Despite this intensive clinical protocol, the prognosis for patients remains very poor with a median survival of 15 months according to tumor radio- and chemo-resistance [2]. Treatment failure may be explained by the presence of highly radioresistant Glioblastoma Stem Cells (GSCs) [3–5]. This small tumor subpopulation shares properties with “normal” neural stem cells like self-renewal activity and multilineage differentiation but shows strong tumorogenicity upon orthotopic transplantation in immunodeficient mice. GSCs represent a supplemental degree in resistance to treatment as they are less sensitive to radiotherapy and contribute to tumor radioresistance by preferential activation of DNA damage checkpoint responses and increased DNA repair capacity [6–9]. Several signaling pathways have been suggested as potential targets in cancer radioresistance including PI3K/Akt, NF-κB, TGF-β, Notch, or STAT3 [10–14]. The transcription factor STAT3 has been shown to play a critical role in GSCs [15–18]. In 2009, Sherry *et al.* have shown for the first time that STAT3 was required for proliferation and maintenance of multipotency in GSCs [19]. This member of STAT (Signal Transducer and Activator of Transcription) family can be activated by various cytokines and growth factors like IL-6 and EGF as well as by oncogenic proteins such as Src and Ras [20–23]. STAT3 is canonically activated by phosphorylation of tyrosine 705 (pY705) by different tyrosine kinases including EGFR, Src, JAK or ERK [24–26]. STAT3 transcriptional activity can be modulated by phosphorylation of serine 727 (pS727) by various serine kinases like PKC, MAPKsor mTOR [27–30]. The activation of STAT3 in the cytoplasm leads to its dimerization by SH2 domains, translocation into the nucleus, DNA binding and transcriptional activation of genes involved in numerous biological processes. Indeed, STAT3 is implicated in inflammatory response, cell proliferation, angiogenesis and cell survival by regulation of anti-apoptotic gene expression such as *Bcl-2* [31–34]. Constitutive activation of STAT3 is frequently found in cancers including GBM [35, 36]. Furthermore, recombinant Erythropoietin Receptor, non-receptor tyrosine kinase BMX, Enhancer of Zeste Homolog 2 or Toll-like receptor 9 were shown to promote GSC self-renewal through activation of STAT3 [37–40]. STAT3 was also shown to be constitutively activated in GSCs and its inhibition impaired GSCs self-renewal and viability [18]. Finally, STAT3 was shown to be involved in radioresistance in breast cancer, colorectal cancer, and gliomas [41–43]. Recently, Ouedraogo *et al.* have shown that STAT3 inhibition by Gö6976 leads to radiosensitization of human conventional GBM cell lines [14]. In this present work, we assessed radiosensitization of patient-derived GSC lines by specific inhibition of STAT3 phosphorylation using Stattic, a small non-peptidic inhibitor of SH2 domain and using WP1066 preventing downstream activation of STAT3 [44, 45]. We also examined STAT3 phosphorylation status on 61 GBM clinical samples to evaluate the prognostic impact of pS727 and pY705.

## RESULTS

### Inhibition of STAT3 phosphorylation affects GSC viability

**Figure 1:**
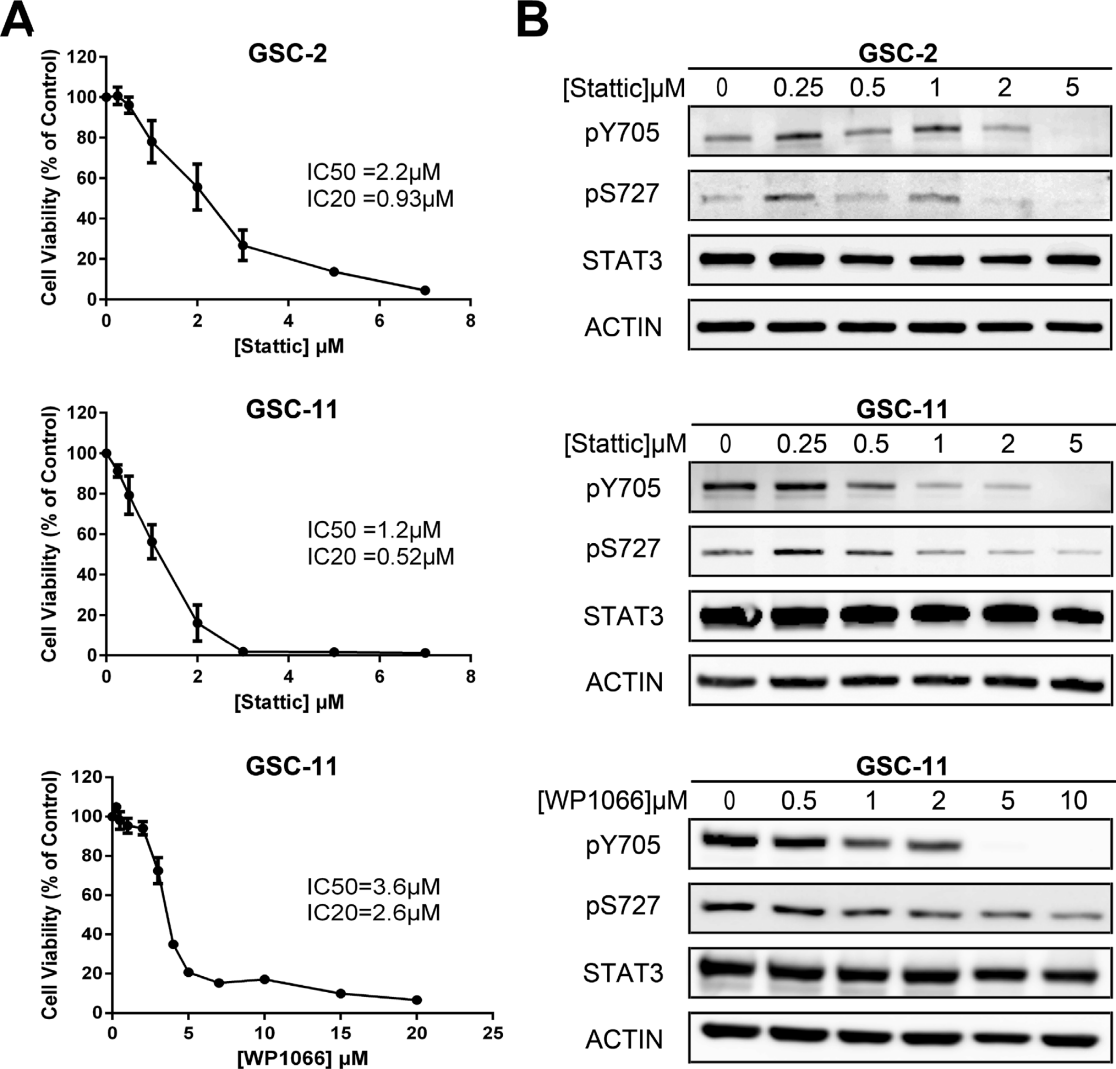
Effect of STAT3 inhibition in GSC lines (**A**) GSC viability was assessed by MTS assay after 5 days of Stattic or WP1066 treatment. The half maximal inhibitory concentrations IC50 and IC20 are indicated in the panel. Each point represents the mean of at least 3 independent experiments. Error bars show ± the standard error of the mean. (**B**) Western Blot analysis of STAT3 phosphorylation status after treatment with indicated doses of each inhibitor. No major modification of total STAT3 expression was observed. Actin was used as internal control. This experiment was repeated 3 times.

As STAT3 is strongly activated in several cancer cell types [46–48], we compared STAT3 activation in our GSCs with normal human Neural Stem Cells (H9-hNSC). We observed that STAT3 is constitutively phosphorylated on both Y705 and S727 in GSCs compared to H9-hNSC (Supplementary Figure 1). As STAT3 is a key player in GSC proliferation and self-renewal, we examined the effect of its inhibition on GSC viability by MTS assay. We observed that Stattic inhibits cell proliferation of both GSC-2 and GSC-11 lines in a dose-dependent manner (Figure 1A). Half-maximal inhibitory concentration (IC50) was 2.2 µM and 1.2 µM whereas IC20 was 0.93 µM and 0.52 µM for GSC-2 and GSC-11 respectively. WP1066 treatment was less toxic compared to Stattic as IC50 and IC20 were 3.6 µM and 2.6 µM for GSC-11. As previous reports showed that Stattic can alter cell cycle distribution, we verified whether Stattic could affect GSC cell cycle [49, 50]. As shown in Supplementary Figure 2, 5 µM of Stattic does not significantly affect the percentage of cells in each phase of the cell cycle. Finally, we examined the effect of Stattic and WP1066 on pY705 and pS727 by western blotting and showed a strong decrease of both phosphorylations (Figure 1B).

### Radiations increase S727 but not Y705 phosphorylation of STAT3

**Figure 2:**
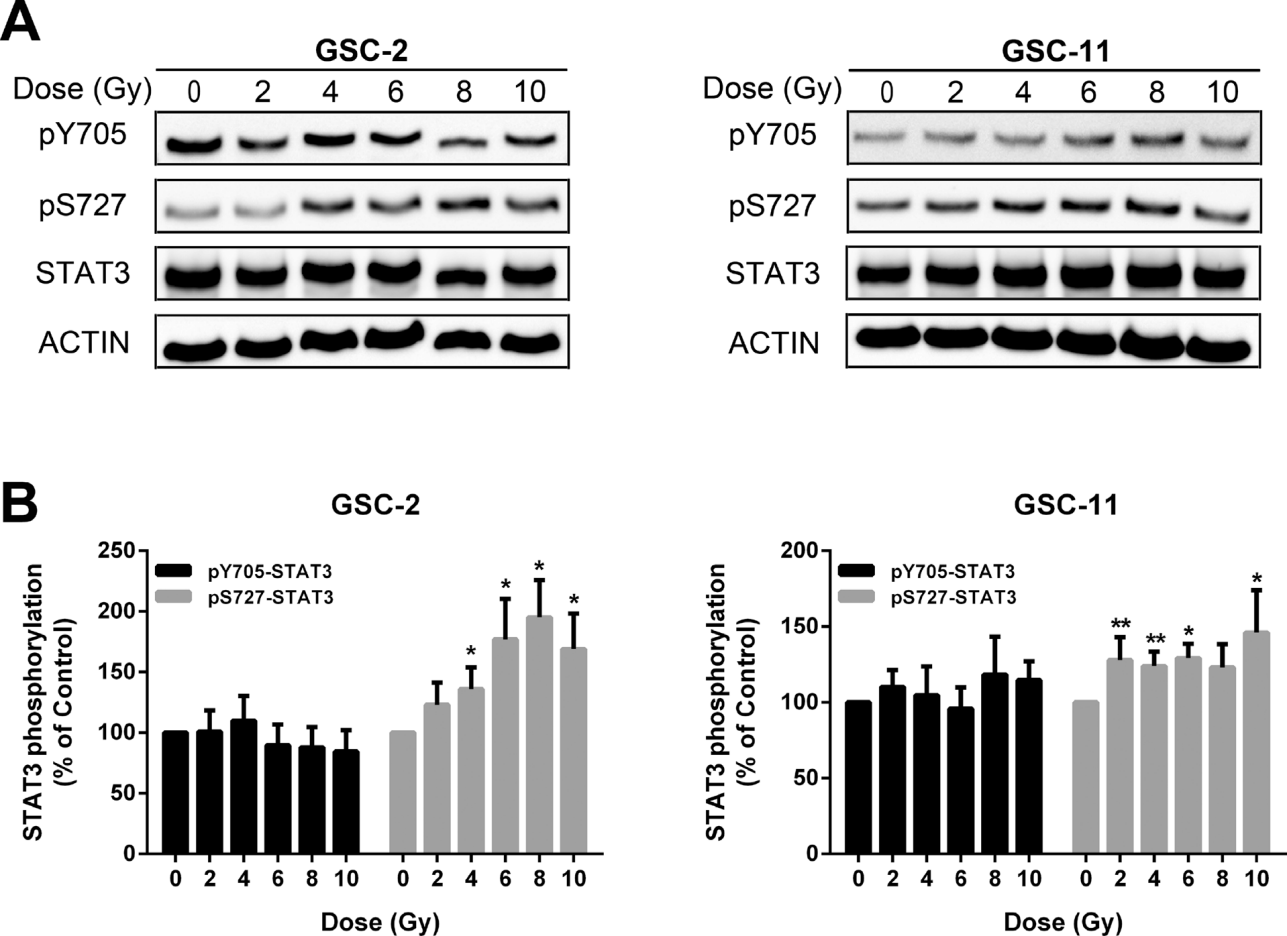
Radiations enhance STAT3 activation mainly by S727 phosphorylation (**A)** Western blot analysis of STAT3 phosphorylation status 24 h after treatment at increasing doses of radiations in GSC-2 and GSC-11. (**B**) Quantification of experiment presented in (A). Histogram represents the mean ± standard error of the mean of 6 independent experiments (^*^p < 0.05; ^**^p < 0.01; Mann-Whitney test).

Several studies have shown that radiations increase STAT3 phosphorylation in tumoral cells [51, 52]. To address the effects of radiations on STAT3 phosphorylation, we irradiated GSC-2 and GSC-11 cell lines at different doses. Lysates of the above cell lines were extracted and the phosphorylation levels of STAT3 on S727 and Y705 were analyzed by Western Blotting (Figure 2A). Twenty-four hours after irradiation, we observed a significant increase of pS727 in both cell lines compared to pY705 (Figure 2B). These results support the idea that irradiation leads to STAT3 activation which may enhance GSC radioresistance.

### Stattic pretreatment radiosensitizes GSCs

**Figure 3:**
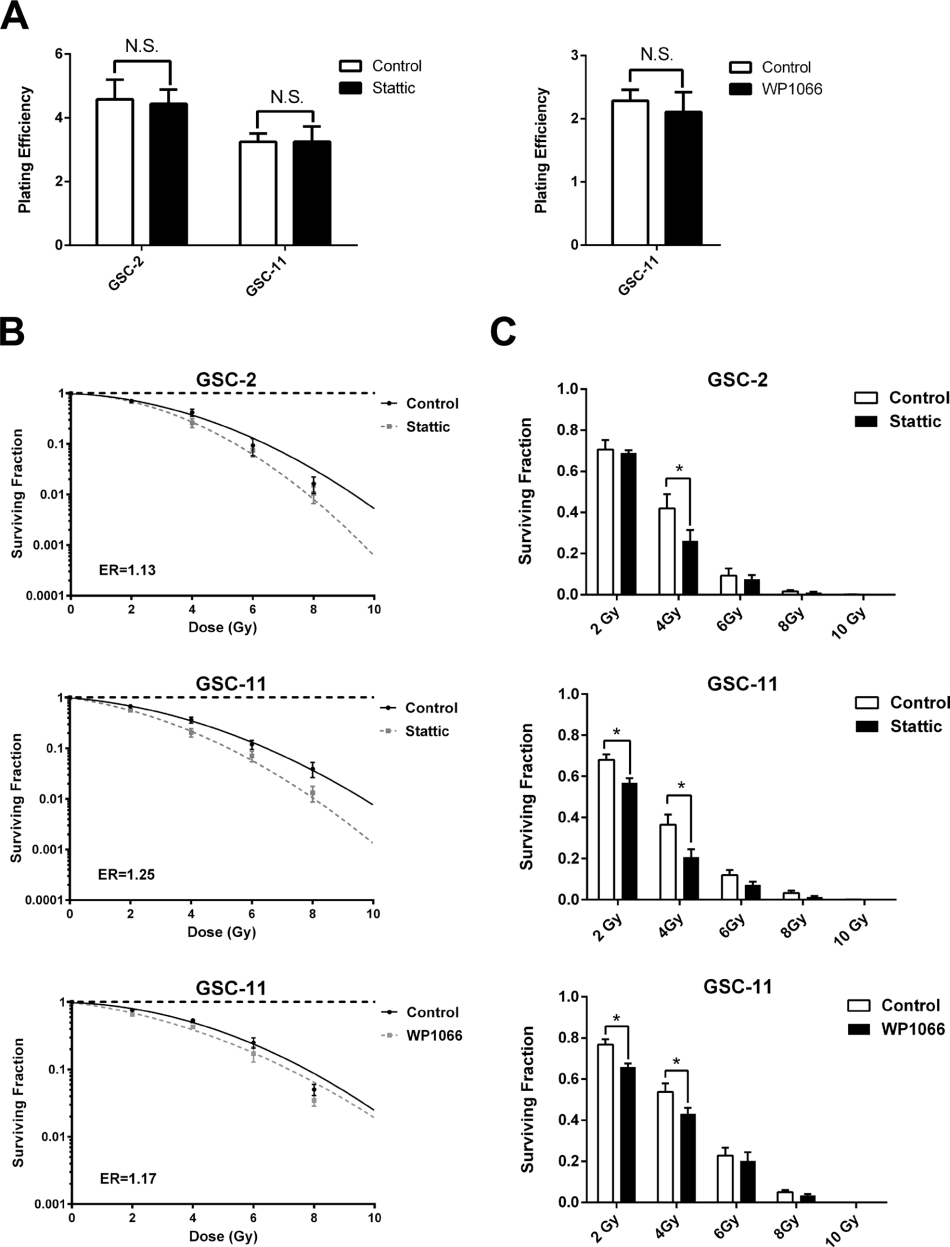
STAT3 inhibition radiosensitizes GSCs (**A**) Plating efficiency of GSC lines in control conditions compared with Stattic or WP1066 pretreatment in the absence of irradiation (N.S. not significant, Mann-Whitney test). (**B**) Clonogenic survival curves of GSCs treated with irradiation alone or in combination with STAT3 inhibitors were assessed using the linear quadratic model. The mean inactivation dose equal to the area under the survival curves was calculated and the cell survival enhancement ratio (ER) was determined as the ratio of the mean inactivation dose under control conditions divided by the mean inactivation dose after inhibitor treatment.A ratio superior to 1 indicates radiosensitization of GSCs. (**C**) Comparison of cell surviving fractions after inhibitor treatment for each dose of radiation. Histograms represent the mean ± standard error of the mean of 4 independent experiments (^*^p < 0.05, Multiple comparison *t*-test).

Since irradiation induced STAT3 activation, we examined whether STAT3 inhibition by Stattic may have a radiosensitizing effect on GSCs. For that purpose, GSC-2 and GSC-11 cell lines were pretreated or not (control) with infra-cytotoxic concentration of Stattic or WP1066 (<IC20). Pretreatment with STAT3 inhibitors at low doses has no effect on cells’ ability to form colonies as evaluated by a clonogenic assay 21 days after plating (Figure 3A). As shown in Figure 3B, radiations combined with Stattic pretreatment drastically decreased the surviving fraction of both GSC lines compared to radiation alone, thereby indicating a radiosensitization effect of STAT3 inhibition (ER > 1). More interestingly, when radiations were associated with Stattic, the effect was found to be statistically significant (*p* < 0.05) at low radiation doses of 2 and 4 Gy for GSC-11 and 4 Gy for GSC-2 (Figure 3C). Additionally, radiosensitization effect of STAT3 inhibition was confirmed on GSC-11 using WP1066 treatment. Thus, we concluded that infra-cytotoxic doses of Stattic or WP1066 potentiate radiation-induced self-renewal inhibition of GSC lines.

### STAT3 preferential activation by pS727 is related to GBM patient outcome

**Figure 4:**
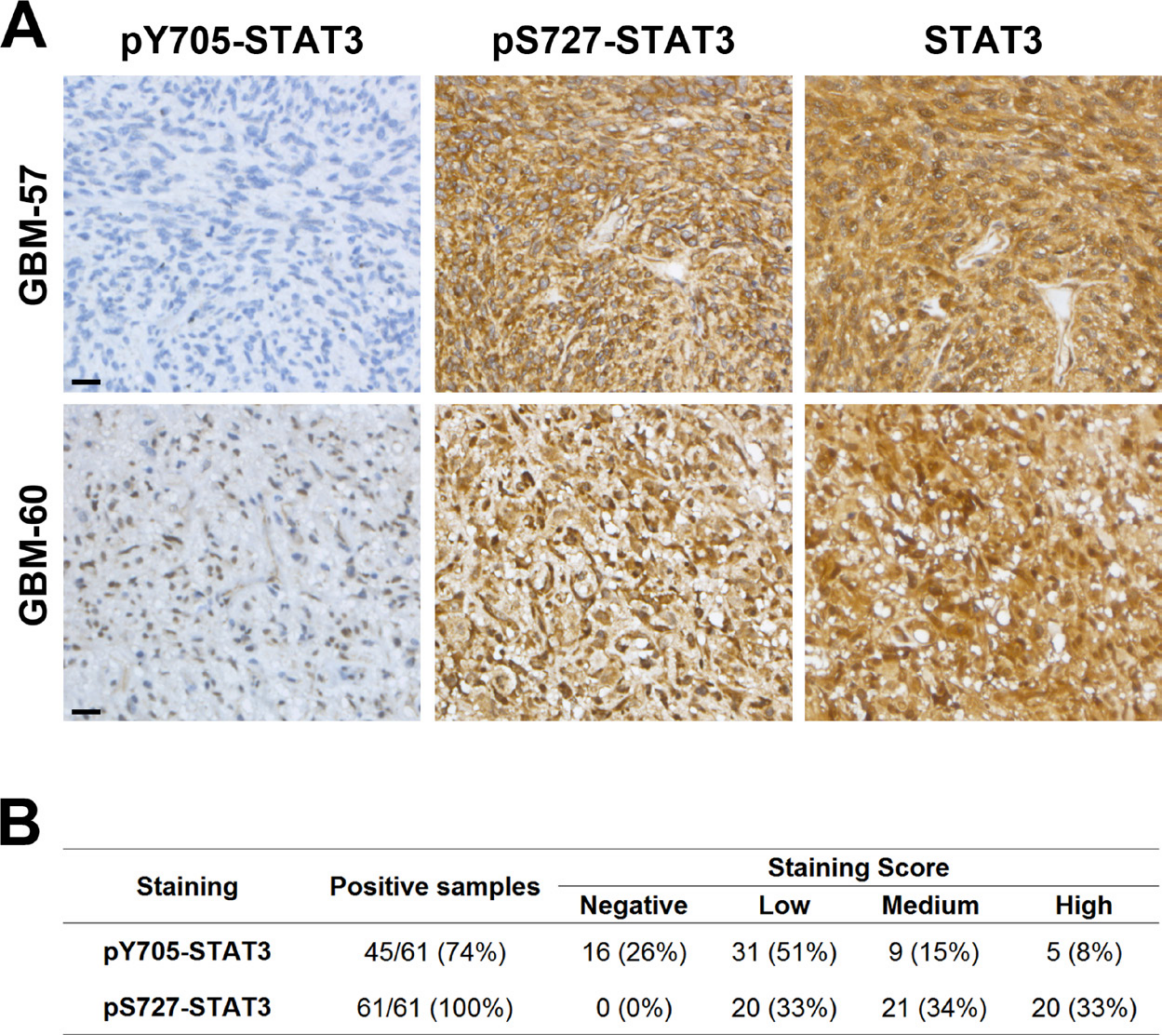
STAT3 is preferentially activated by pS727 in GBM clinical samples (**A**) Examples of immunohistochemical staining to detect the phosphorylation of Y705 and S727 or the expression of total STAT3 in 2 GBM samples. Scale bar = 20 µm. (**B**) Number and percentage of positive samples and staining score for pY705 and pS727 (*n* = 61 patients).

**Figure 5:**
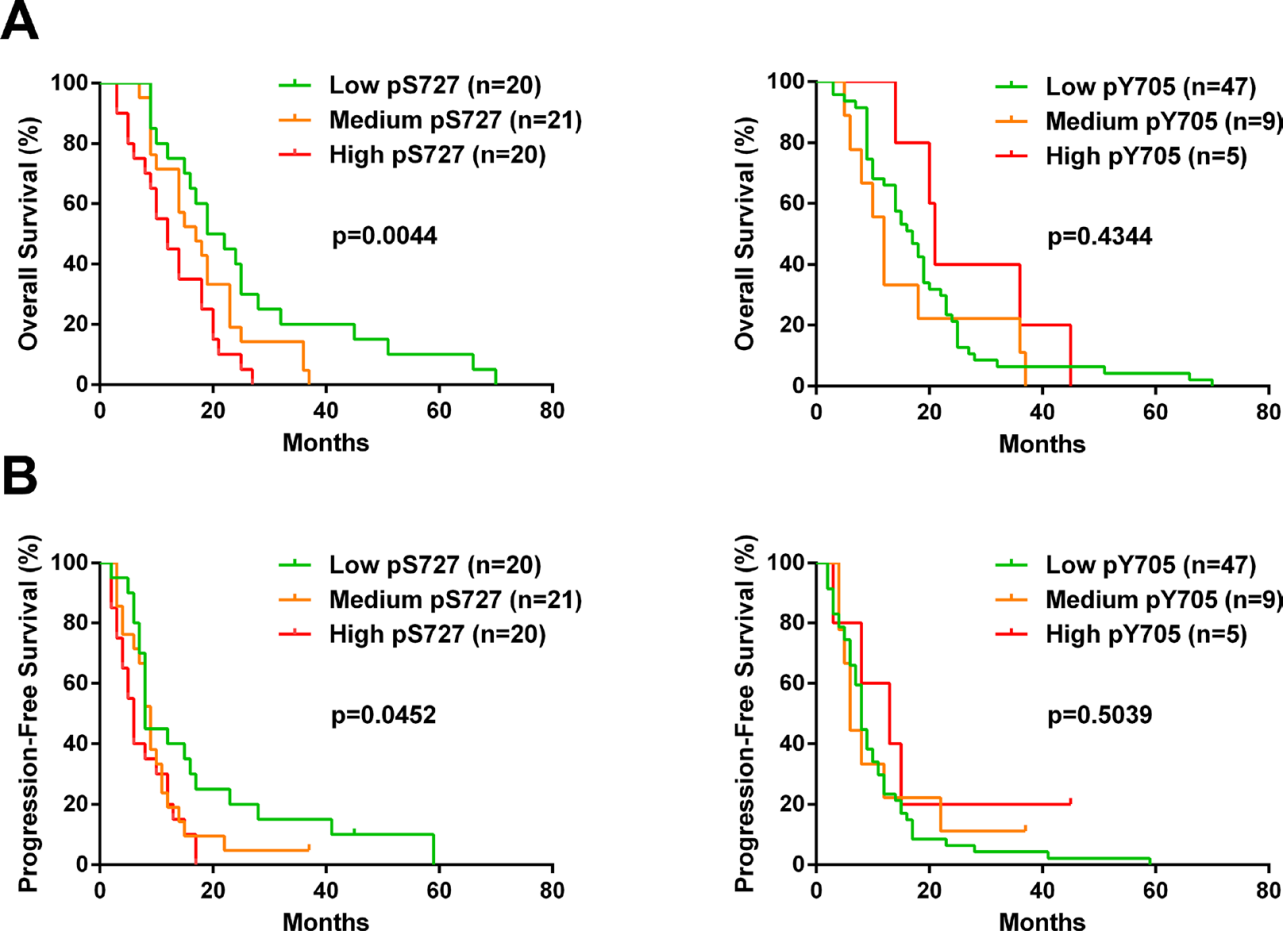
S727 phosphorylation but not Y705 affects the outcome of GBM patients Kaplan-Meier curves of all glioblastoma patients plotting overall survival (**A**) or progression-free survival (**B**) of patients with low, medium or high score for pY705 and pS727 staining. For this analysis, patients presenting negative or low staining score were pooled. Mantel-Cox log rank test was performed to determine the *p*-value indicated on the graphs.

To investigate the activation pattern of STAT3 in clinical samples, we assessed pY705, pS727 and total STAT3 by immunohistochemistry (IHC) on Tissue Microarray comprising samples of 61 patients with GBM (Figure 4A). First, we found that all samples were positive for pS727 whereas 74% of these samples showed pY705 staining thereby suggesting an important role of pS727 activation in GBM (Figure 4B). Moreover, 77% of samples presented a low or negative staining for pY705 and only 8% were associated with a high level staining. On the contrary, concerning pS727 immunolabeling, 33% of the above cases exhibited low staining levels whereasile 33% were highly stained. Finally, we evaluated the prognostic role of pS727 and pY705 on our patient’s cohort. The analysis of Kaplan-Meier survival curves showed that a high level of pS727 was associated with a lower overall survival (*p* = 0.0044) and progression-free survival (*p* = 0.0452) whereas no correlations were found for pY705 (*p* = 0.4344 and *p* = 0.5039 respectively) (Figure 5A and 5B). Taken together, these data suggest that pS727-STAT3 has a prognostic value and could be involved in GBM aggressiveness and resistance.

## DISCUSSION

Concomitant chemo and radiotherapy after surgery represent the “gold standard” for initial treatment of GBM. Despite this aggressive therapy, relapse ineluctably occurs, principally due to tumor resistance. This therapeutic failure may be explained by the presence of highly radioresistant GSCs which constitute an additional degree of resistance and could be one of the main causes of tumor relapse. Many avenues have been suggested to target pathways involved in gliomagenesis and therapeutic resistance in order to increase treatment efficiency and patient survival. Transcriptional factor STAT3 was proposed as a potential target for tumor radiosensitization. Indeed, STAT3 is a major actor of cell survival after therapy by regulating the expression of anti-apoptotic genes such as *Bcl-xL* or *Mcl-1* in glioblastoma [53]. Moreover, STAT3 was shown to be necessary for efficient repair of damaged DNA, partly by modulating the ATM-Chk2 and ATR-Chk1 pathways [54]. Recently, Xu *et al.* have shown that autophagy promotes the repair of radiation-induced DNA damage in bone marrow hematopoietic cells through the activation of STAT3, leading to upregulation of expression of *BRCA1* [55]. Furthermore, several studies have demonstrated that the inhibition of STAT3 led to radiosensitization of different cancer cell lines including breast, colorectal, uterine, head and neck and brain cancers [14, 41, 42, 56, 57]. In 2016, Ouédraogo *et al.* proposed that pS727-STAT3 constitute a relevant target for radiosensitization in human GBM cell lines. However, radiosensibility after STAT3 inhibition was only observed for conventional cell lines which present pS727 without pY705 [14].

In this work, we investigated the radiosensitizing effect of STAT3 inhibition by Stattic in two patient derived GSC lines and assessed the prognostic impact of STAT3 activation in 61 GBM clinical samples. First, we showed that STAT3 is constitutively activated in our GSC lines confirming our previous report [18]. Moreover, this activation is increased by radiation treatment mainly on pS727. Stattic inhibitor induces a strong decrease of both pY705 and pS727 phosphorylations in GSCs. For determination of conjugated effect of STAT3 inhibition with radiations, we used infra-cytotoxic dose (<IC20) of Stattic to exclude inhibitory effect alone. For both GSCs, Stattic potentiated radiation effect by decreasing GSC self-renewal. This effect was more important for low radiation doses at 2 and 4 Gy corresponding to daily fractions of radiotherapy received by GBM patients. We also confirmed the strategy of STAT3 inhibition for GSC radiosensitization using a second inhibitor, WP1066. Our results showed that STAT3 inhibition led to radiosensitization even in the presence of both STAT3 phosphorylated forms in opposition to previous results reported by Ouédraogo *et al.* However, these results corroborate the fact that S727 phosphorylation constitutes an interesting target to sensitize GSCs to irradiation.

Over the last decade several STAT3 inhibitors have been studied but Stattic has been the first nonpeptidic small molecule to demonstrate selective inhibition function over the STAT3 SH2 domain regardless of the STAT3 activation state *in vitro* [44]. Other STAT3 inhibitors are currently used for targeting the STAT3 pathway in cancer, including GBM. Cucurbitacin-I and WP1066 administration or shRNA knockdown resulted in on-target JAK2/STAT3 inhibition and dramatically reduced GBM-derived Brain tumor stem cells [17]. Moreover, STX-0119 inhibitor of STAT3 dimerization has shown antitumor effects on GSC lines without significant decrease of pY705 [58]. WP1066, one of the most promising STAT3 inhibitor, will be investigated in phase I clinical trial for patients with recurrent malignant glioma and brain metastasis from melanoma (scheduled for 2017, ClinicalTrials.gov). As WP1066 is orally bioavailable and crosses the blood-brain barrier, it could be a promising inhibitor of STAT3 in clinical treatment of GBM [59]. OPB-31121 is another promising STAT3 inhibitor which presents notably higher affinity for STAT3, stronger efficacy for pY705 and pS727 inhibition and is associated with a lower toxicity than other STAT3 inhibitors. This inhibitor induces a strong decrease of cell proliferation and clonogenicity in prostate cancer cell lines [60]. In addition, OPB-31121 has been tested in a phase I clinical trial, showing antitumoral activity with relatively good tolerance and demonstrating feasibility of STAT3 inhibition in patients with solid tumors [61]. Altogether, these data demonstrated the potential role of STAT3 inhibitors in cancer treatment, however these molecules cannot distinguish which activation of STAT3 could be at the origin of tumor resistance as there are no pS727 specific inhibitors.

In our study, we observed that GSC lines showed different sensibility in term of response to Stattic treatment with almost a 2 fold change in IC50 and IC20 values. This could be explained by tumor heterogeneity with distinct genetic profiles. Indeed, GBMs were classified into four different groups (classical, mesenchymal, neural and proneural) according to their molecular and transcriptome profiles referred to as Verhaak’s classification [62]. These subtypes present different microenvironments and are regulated by different signaling pathways. The mesenchymal subtype was shown to be particularly malignant and associated with a poor prognosis [63]. In brain tumors, STAT3 was shown to play a critical role in mesenchymal transition associated with angiogenesis, extensive necrosis and enhanced inflammatory response [15, 64–66]. The experimental differences between our GSC lines could be explained by inter and intra-tumor heterogeneity.

Immunohistochemical analysis of pS727 and pY705 in our 61 GBM samples showed that pS727 was present in all patients with higher staining scores than pY705. Moreover, we found that pS727 negatively impacts patient outcome in a way that is concordant with previous reports showing the association of pS727 with lower patient survival and faster relapse [14, 67]. However, we did not find any association for pY705 in contrast to previous studies which have also shown a prognostic role for pY705 [68]. This could be explained by the limits of sensitivity and specificity of the diagnosis and of the detection methods as well as by the sampling and monitoring of patients. In our cohort, all patients were deceased at the time of the study removing bias due to censored subjects in Kaplan-Meier survival curve analysis.

In conclusion, our results show pS727 as prognostic factor for patient survival and confirm the strategy of GSC radiosensitization by STAT3 inhibition. The high level of pS727 could confer a radioresistance of GBM tumor and explain faster relapse after current radio-chemotherapy treatment, supporting the idea of pS727-STAT3 inhibition as innovative targeted therapy. However, genetic background of tumor could modulate the therapeutic response, suggesting that the strategy to enhance tumor radiosensitivity by STAT3 inhibition could be applied to STAT3-dependant GBMs, especially these of the mesenchymal subtype.

## MATERIALS AND METHODS

### Cell culture

Glioblastoma stem cell cultures were derived from adult patients with high-grade gliomas operated in the University Hospital of Poitiers after informed consent of all patients as previously described [9, 18, 69, 70]. Briefly, both GSC lines were assessed for stemness, self-renewal and differentiation *in vitro*, then tumorigenicity was evaluated by xenografts in immunodeficient mice. Cells were cultured in Neurobasal^®^ Medium (NBE) (Gibco^®^) supplemented with B27 1%; N2 0.5%; bFGF 0.05% and EGF 0.005% (Gibco^®^) at 37°C in 5% CO_2_ humidified incubator. Culture medium was replaced twice a week and when spheres became large and numerous, they were enzymatically dissociated with accutase (Merck Millipore).The molecular characteristics of the GSC-2 and GSC-11 including MGMT promoter methylation, EGFR copy number, IDH1, IDH2, EGFR-variant III, p53, PTEN status as well as LOH at loci 1p36, 19q13, 9p21 and 10q23 are provided in supplemental table S1. Human Neural Stem Cells (H9-NSCs) (Gibco^®^) were cultured following the manufacturer’s instructions.

### Cell viability assay

The MTS cell test (CellTiter 96^®^ Aqueous Non-Radioactive Cell Proliferation Assay (Promega)) was used to determine cell viability. Cells were seeded in a 96-well plates at 50 000 cells per plate and treated one day after with appropriate dose of Stattic (Merck Millipore) or WP1066 (Santa Cruz Biotechnology). Five days later, quantification of viable cells was performed at 492 nm with a micro plate reader (Dynex Technologies, Chantilly, France).

### Cell irradiation

Cell irradiation was performed at the Department of Radiotherapy of the University Hospital of Poitiers. Cells were submitted to gamma irradiation with a linear accelerator Elekta Synergy Beam Modulator (4.56 Gy/min).

### Western blot

Cells were lysed 7 h after Stattic treatment, 6 h after WP1066 treatment or 24 h after irradiation with a cold RIPA buffer and protein concentration was determined using a Bradford assay (Bio-rad). An equal quantity of protein samples was separated by SDS-PAGE and transferred onto a nitrocellulose membrane (Bio-rad). Membrane was blocked with Phosphate-buffered saline (PBS 1X) containing 0.1% Tween 20 (Sigma) and 5% non-fat dry milk. Primary antibodies were incubated overnight at 4°C (Stat3 1:1250 (#9136); phosphor-Stat3 Tyr705 1:500 (#9131); phosphor-Stat3 Ser727 1:500 (#9136) Cell Signaling Technology; Actin 1:5000 (#ab3280) Abcam). Secondary antibodies were incubated for 1h 30 at room temperature (Anti-mouse HRP-linked 1:2000 (#7076); Anti-rabbit HRP-linked 1:2000 (#7074) Cell Signaling Technology). Immunoblotting signals were detected using an enhanced chemiluminescence method (Clarity^™^ Western ECL Substrate, Bio-rad) with Luminescent Image Analyzer LAS-3000 (FUJIFILM). Densitometry analyses were performed using ImageJ software (imagej.nih.gov/ij/). Relative amounts of pY705 and pS727 were normalized to total STAT3 and Actin.

### Clonogenic assay

Neurospheres were dissociated and treated with appropriate doses of Stattic (<IC20; 0.5 µM for GSC-11 and 0.25 µM for GSC-2) or WP1066 (<IC20; 1.5 µM) for 7 h and 6 h respectively prior to irradiation. Cells were plated in triplicate at a density of 4 × 10^4^ cells per 35 mm dish in methylcellulose medium (Neural Cell Cloning Medium-S1, Stem Cells Technology) supplemented with B27 1%; N2 0.5%; bFGF 0.05%; EGF 0.005% and subjected to various doses of irradiation (2, 4, 6, 8 and 10 Gy). After 21 days of incubation, formed colonies were counted under inverted microscope (Nikon). Survival curves were obtained using linear-quadratic model: Surviving Fraction = *e*^*–α(D + βD2)*^. The mean inactivation dose equal to the area under the survival curves was calculated and the cell survival enhancement ratio (ER) was determined as the ratio of the mean inactivation dose under control conditions divided by the mean inactivation dose after inhibitor treatment. A ratio superior to 1 indicates radiosensitization of GSCs.

### Tissue Microarray (TMA) construction, immunohistochemistry and scoring

TMAs were constructed using formalin-fixed paraffin embedded tissue samples that represent a total of 61 GBM from surgical resection or biopsy patients operated at the University Hospital of Poitiers. Patient characteristics are summarized in supplemental table S2. All of these patients were treated with radiotherapy and temozolomide. Original slides were reviewed to confirm GBM histology according to the 2007 World Health Organization classification system. For each case, a minimum of 3 cores were transferred from the selected areas to the recipient block, using a TMA workstation (Alphelys, Plaisir, France). The recipient block was cut into 3 μm thick section, and immunochemistry was performed with an automated system (BenchMark XT, Ventana, Roche). Briefly, slides were deparaffinized and heated in sodium citrate pH6 solution for antigenic retrieval. Primary antibodies were incubated overnight at 4°C (phospho-Stat3 Tyr705 1:100 (#9131); phospho-Stat3 Ser727 1:200 (#9136); Stat3 1:1250 (#9136) Cell Signaling Technology) and revealed using the streptavidin-biotin-peroxidase method with diaminobenzidine as chromogen (UltraView universal DAB detection kit, Roche). Scoring of antibody staining was evaluated independently by two pathologists in a blind manner. For both pY705 and pS727 staining, the percentage of stained tumor cells was multiplied by staining intensity (weak = 1, moderate = 2 or strong = 3) to get a score between 0 and 300. Patient samples were categorized according to the statistical distribution of staining scores into 3 groups: Low, Medium or High. Survival rates were estimated by the Kaplan–Meier method.

### Cell cycle analysis

GSCs were treated with 5 μM of Stattic during 7 hours. Then, cells were fixed with 70% ethanol at –20°C, washed once with cold PBS 1X and resuspended in DNA staining solution (2.5 µg/ml Propidium Iodide, 0.5 mg/ml RNase A) (MerckMillipore). Cell cycle redistribution was measured by flow cytometry on a FACS Canto II (BD Biosciences). Data analysis was performed using FlowJo^®^ software (LLC). A total of 10 000 events were analyzed in 4 independent experiments.

### Statistical analysis

Descriptive statistics of the results were calculated with GraphPad Prism 6 (La Jolla, CA, USA). All experiments were performed at least three times. The results are presented as mean ± standard error of the mean (SEM) and statistical significance was evaluated by Mann-Whitney test or multiple comparison *t*-test (^*^p < 0.05). Log-rank (Mantel-Cox) test was applied to Kaplan-Meier survival curves and exact p-values were indicated.

## SUPPLEMENTARY MATERIALS FIGURES AND TABLES





## Abbreviations

Bcl-2: B-cell lymphoma 2
EGFR: Epidermal Growth Factor Receptor
ER: enhancement ratio
ERK: extracellular signal–regulated kinase
GBM: Glioblastoma
GSCs: Glioblastoma Stem Cells
IHC: immunohistochemistry
JAK: Janus kinase
PI3K: MAPKs: Mitogen-activated protein kinases
mTOR: mechanistic target of rapamycin; Phosphoinositide 3-kinase
PKC: Protein kinase C
STAT3: Signal Transducer and Activator of Transcription 3
TGF-β: Transforming growth factor beta
TMA: Tissue Microarray
