# Supplementary material for: Impact of STAT3 phosphorylation in glioblastoma stem cells radiosensitization and patient outcome
Source: Oncotarget. 2017 Dec 16;9(3):3968–79. doi: 10.18632/oncotarget.23374 (PMC5790515; doi:10.18632/oncotarget.23374)
Supplement: Supplementary file 1 [file oncotarget-09-3968-s001.pdf]

Impact of STAT3 phosphorylation in glioblastoma stem cells radiosensitization and patient outcome

SUPPLEMENTARY MATERIALS

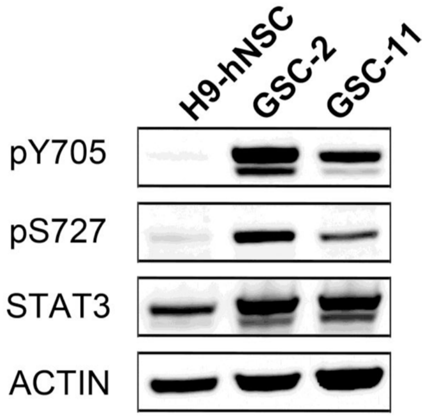

Supplementary Figure 1: STAT3 phosphorylation status in GSC lines compared to normal human Neural Stem Cells (H9-hNSC).

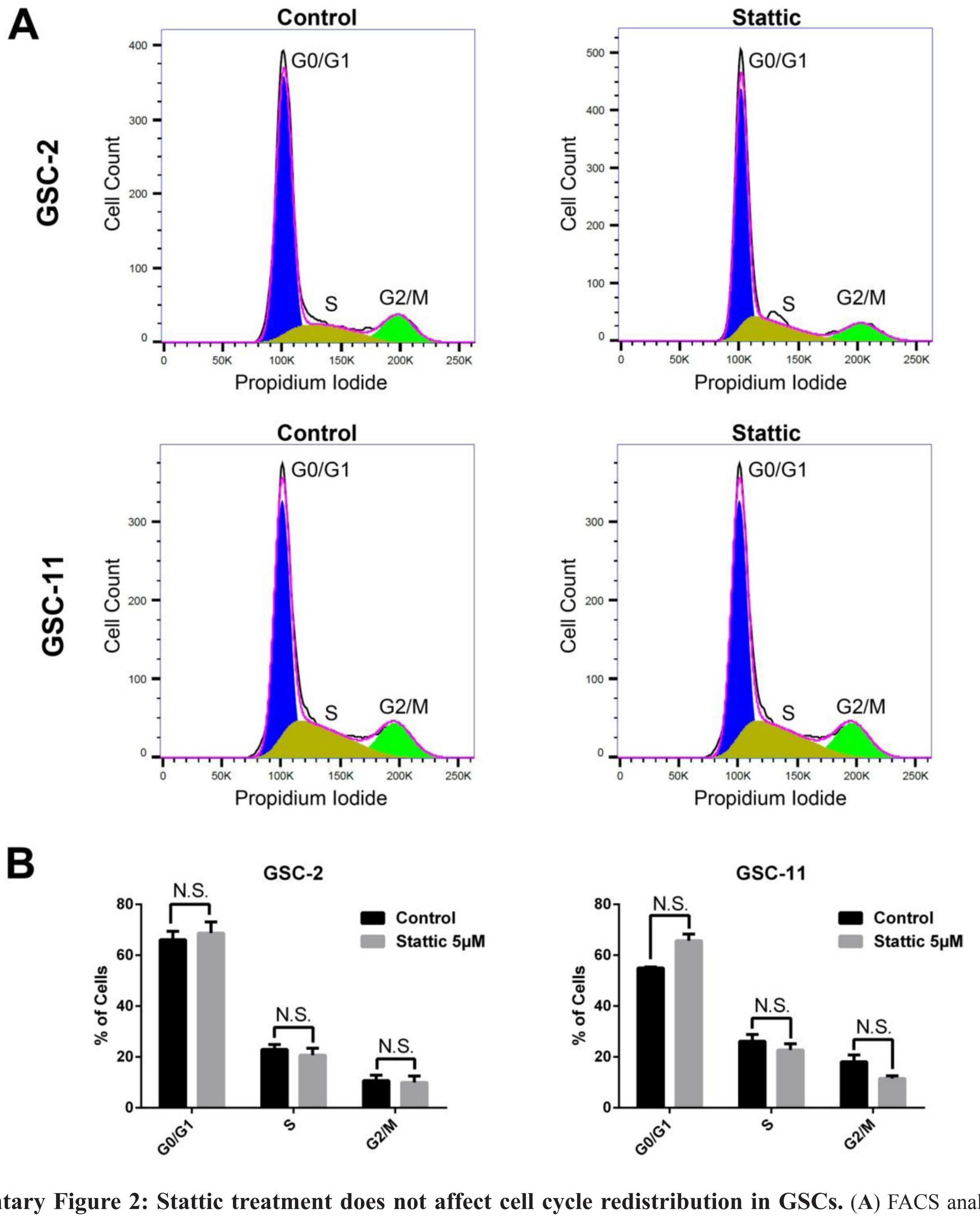

Supplementary Figure 2: Stattic treatment does not affect cell cycle redistribution in GSCs. (**A**) FACS analysis of cell cycle in GSC-2 and GSC-11 with or without Stattic treatment. (**B**) Quantification of experiment presented in (A). Histogram represents the mean ± standard error of the mean of 4 independent experiments (N.S. not significant, Mann-Whitney test).

Supplementary Table 1: Molecular characteristics of GSC-2 and GSC-11

GSC line	GSC-2	GSC-11
**IDH1**	WT	WT
**IDH2**	WT	WT
**p53**	WT	WT
**PTEN**	Mut	WT
**EGFRvIII**	NO	YES
**EGFR amplification**	polysomy	polysomy
**LOH 1p36**	NO	NO
**LOH 19q13**	NO	NO
**LOH 9p21**	YES	N.D.
**LOH 10q23**	YES	N.D.
**MGMT methylation (%)**	1.6	94.2

Supplementary Table 2: Clinical data of 61 GBM patients. See Supplementary_Table_2
